# A microbiological study of neonatal conjunctivitis in two hospitals in Tehran, Iran

**DOI:** 10.1186/1471-2334-12-S1-P48

**Published:** 2012-05-04

**Authors:** Seyed Abolfazl Afjeiee, Sedigheh Rafiei Tabatabaei, Fatemeh Fallah, Arezou Tavakkoly Fard, Farideh Shiva, Saadat Adabian, Abdollah Karimi

**Affiliations:** 1 Pediatric Infectious Research Center, Mofid Children Hospital, Shahid Beheshti University of Medical Sciences, Tehran, Iran; 2 Department of Neonatal Disease, Mahdieh Hospital, Shahid Beheshti University of Medical Sciences, Tehran, Iran

## Background

Conjunctivitis during the neonatal period is accompanied by diffuse conjunctival injection; it is usually acquired and may result in serious eye damage. This study is to define the prevalence of neonatal conjunctivitis and to identify the causative agents of ophthalmia neonatorum in two university hospitals from 2008-2009.

## Methods and materials

All neonates admitted in the neonatal department during the study period were examined for the presence of conjunctivitis. Two swab specimens containing epithelial cells of the conjunctiva were collected from newborns presenting with conjuntival inflammation. Laboratory diagnosis was based on bacterial culture and Gram staining. The isolated bacteria were identified using standard procedures. For identifying *Chlamydia trachomatis* we used PCR and cell culture.

## Results

Of the 2253 neonates, (age range 1-30 days), clinical findings of conjunctivitis were found in 241 cases, (10.7%). The most commonly isolated bacteria were Coagulase Negative *Staphylococci*, (N=130, 53.9%); *Chlamydia trachomatis* was the second most common cause of acute neonatal conjunctivitis, (n=40, 16.6%). Bacterial cultures were negative in 47 neonates (19.5%) despite clinical signs of conjunctivitis. The median age at presentation for bacterial culture positive was day 8 of life.

## Conclusion

Neonatal conjunctivitis is prevalent in newborns; Gram Positive Cocci and *Chlamydia trachomatis* are the most common causative organisms.

